# Hospital Admission Trends Due to Viral Infections Characterised by Skin and Mucous Membrane Lesions in the Past Two Decades in England and Wales: An Ecological Study

**DOI:** 10.3390/ijerph182111649

**Published:** 2021-11-05

**Authors:** Abdallah Y. Naser, Hamzeh Mohammad Alrawashdeh, Hassan Alwafi, Amal Khaleel AbuAlhommos, Zahraa Jalal, Vibhu Paudyal, Zahra Khalil Alsairafi, Emad M. Salawati, Mohammed Samannodi, Kanar Sweiss, Yousef Aldalameh, Fatemah M. Alsaleh, Mohammad Abusamak, Ahmad Shamieh, Eyad I. Tantawi, Mohammad S. Dairi, Motaz Dairi

**Affiliations:** 1 Department of Applied Pharmaceutical Sciences and Clinical Pharmacy, Faculty of Pharmacy, Isra University, Amman 11622, Jordan; 2 Department of Ophthalmology, Sharif Eye Centers, Irbid 11511, Jordan; dr_hmsr@yahoo.com; 3 Faculty of Medicine, Umm Al Qura University, Mecca 21514, Saudi Arabia; hhwafi@uqu.edu.sa (H.A.); samannodi@gmail.com (M.S.); msdairi@uqu.edu.sa (M.S.D.); dr_mgdairi@hotmail.com (M.D.); 4 Alnoor Specialist Hospital, Ministry of Health, Mecca 24241, Saudi Arabia; 5 Pharmacy Practice Department, Clinical Pharmacy College, King Faisal University, Alhasa 43518, Saudi Arabia; aabualhomos@kfu.edu.sa; 6 School of Pharmacy, Institute of Clinical Sciences, University of Birmingham, Birmingham B15 2TT, UK; z.jalal@bham.ac.uk (Z.J.); V.Paudyal@bham.ac.uk (V.P.); 7 Department of Pharmacy Practice, Faculty of Pharmacy, Kuwait University, Kuwait City 12037, Kuwait; zahra.alsairafi@ku.edu.kw (Z.K.A.); fatemah.alsaleh@ku.edu.kw (F.M.A.); 8 Department of Family Medicine, Faculty of Medicine, King Abdulaziz University, Jeddah 21589, Saudi Arabia; Esalawati@kau.edu.sa; 9 Department of Basic Pharmaceutical Sciences, Faculty of Pharmacy, Isra University, Amman 11622, Jordan; kanar.sweiss@iu.edu.jo (K.S.); yousef.dalahmeh@iu.edu.jo (Y.A.); 10 Department of General and Special Surgery, Faculty of Medicine, Al Balqa Applied University, Salt 19117, Jordan; mabusamak@bau.edu.jo; 11 Daniel Castro Dental Clinics, El Paso, TX 79911, USA; Dentist.shamieh@gmail.com; 12 Department of General Surgery, King Faisal Hospital, Ministry of Health, Mecca 11211, Saudi Arabia; Eyadtantawi@gmail.com

**Keywords:** the United Kingdom, England, Wales, hospitalisation, admission rate, skin, mucous membrane, viral infections

## Abstract

Objectives: This study aimed to investigate the trends in hospital admissions due to viral infections characterized by skin and mucous membrane lesions in England and Wales between 1999 and 2019. Methods: This is an ecological study using publicly available databases in England and Wales; the Hospital Episode Statistics database in England and the Patient Episode Database for Wales. Hospital admissions data were collected for the period between April 1999 and March 2019. Hospital admissions due to viral infections characterized by skin and mucous membrane lesions were identified using the tenth version of the International Statistical Classification of Diseases system, diagnostic codes B00–B09. The trend in hospital admissions was assessed using a Poisson model. Results: Hospital admissions for different causes increased by 51.9% (from 25.67 (95% CI 25.23–26.10) in 1999 to 38.98 (95% CI 38.48–39.48) in 2019 per 100,000 persons, trend test, *p* < 0.01). The most prevalent viral infections characterized by skin and mucous membrane lesions hospital admissions causes were zoster (herpes zoster), varicella (chickenpox), herpesviral (herpes simplex) infections, and viral warts, which accounted for 26.9%, 23.4%, 18.7%, and 17.6%, respectively. The age group below 15 years accounted for 43.2% of the total number of admissions. Females contributed to 50.5% of the total number of admissions. Hospital admission rate in males increased by 61.1% (from 25.21 (95% CI 24.59–25.82) in 1999 to 40.60 (95% CI 39.87–41.32) in 2019 per 100,000 persons). The increase in females was 43.2% (from 26.11 (95% CI 25.49–26.72) in 1999 to 37.40 (95% CI 36.70–38.09) in 2019 per 100,000 persons). Conclusion: Our study demonstrates an evident variation in hospital admission of viral infections characterized by skin and mucous membrane lesions based on age and gender. Efforts should be directed towards vaccinating high-risk groups, particularly the elderly and females. Moreover, efforts should be focused on vaccinating the young population against varicella, particularly females who are more susceptible to acquiring the infection. Further observational and epidemiological studies are needed to identify other factors associated with increased hospital admission rates.

## 1. Introduction

A virus is a microscopic organism that cannot live outside the host body [1]. It is composed of either ribonucleic acid (RNA) or deoxyribonucleic acid (DNA) as genetic material [1]. Infectious diseases are a major cause of morbidity and mortality worldwide [2]. There are several types of viral infections, some of which are prevalent, such as those responsible for respiratory and gastrointestinal tract infections [3].

Viruses cause a wide range of infectious diseases and are a major concern for public health. Viral infections pose a significant burden on the healthcare system and the economy, accounting for around 7% of deaths and annual costs of GBP 30 billion [4]. Infections due to viruses are responsible for millions of deaths worldwide [1]. Some viral infections are chronic in nature and are associated with poor health outcomes and significant morbidities, such as infections due to human immunodeficiency virus (HIV) and hepatitis viruses [1]. 

In the last century, improvements in public health concerning sanitation, vaccination programmes such as the national immunisation programme, and other public health interventions aided in reducing the burden of infectious diseases [5,6]. However, viruses are continuously evolving, spreading, and imperceptibly changing their behaviours, so the hospitalisation rate due to viral infectious remains high [2]. In addition, in the last 20 years, several treatments have been approved for critical and common chronic viral infections, such as hepatitis B and C and HIV [7,8]. 

Viruses such as human papilloma virus (HPV) and herpes simplex virus can infect the skin and mucus membrane [9,10]. The mucous membranes, also known as the mucosae, are membranes that line several body cavities, the surfaces of internal organs, and canals leading to the outside of the body, particularly the gastrointestinal, respiratory, and genitourinary tracts. They originate in the endoderm and continue to the skin at body openings, including the internal areas of the mouth, nose, lips, eyes, ears, urethral opening, anus, and vagina [11]. 

Previous studies that examined the trends in hospital admissions due to viral infections characterised by skin and mucous membrane lesions in the UK are limited and restricted to specific skin and mucous membrane diseases such as eczema and bullous pemphigoid [12,13,14] or specific age group [15]. A previous study by Abdalrahman et al. explored the trend of hospital admissions in England for varicella and herpes zoster from 2001 to 2011 and reported that hospital admissions rate for varicella increased by 1.8% during the study period. On the other hand, the same study reported that the overall admission rates for herpes zoster have decreased by 4% [16].

Therefore, in this study, we aimed to investigate the trends in hospital admissions due to these particular viral infections in England and Wales in the past two decades. This will help in identifying key demographic factors that lead to this type of hospital admissions, which ultimately could facilitate the development of prevention measures directed towards high-risk populations.

## 2. Methods

### 2.1. Study Sources and the Population

This was an ecological study using data extracted from the Hospital Episode Statistics (HES) database in England [17] and the Patient Episode Database for Wales (PEDW) for the period between April 1999 and April 2019 [18]. They have been used previously to explore the trends of different health outcomes and the associated hospital admissions [19,20]. The HES and PEDW databases contain hospital admission data for patients with viral infections characterized by skin and mucous membrane lesions from all age groups, which are subdivided into four categories; below 15 years, 15–59 years, 60–74 years, and 75 years and above. We identified hospital admissions using the 10th version of the International Statistical Classification of Diseases (ICD) system. Diagnostic codes (B00–B09) were used to identify all hospital admission related to various types of viral infections characterized by skin and mucous membrane lesions in England and Wales. HES and PEDW databases record all hospital admissions, outpatients, and Accident and Emergency (A&E) activities performed at all National Health Service (NHS) trusts and any independent sector funded by National Health Services (NHS) trusts. HES and PEDW data are checked regularly to ensure their validity and accuracy [17,18,21]. To calculate the annual hospital admission rate for viral infections characterized by skin and mucous membrane lesions, we collected mid-year population data for the period between 1999 and 2019 from the Office for National Statistics (ONS) [22]. 

### 2.2. Statistical Analysis

Hospital admission rates with 95% confidence intervals (CIs) were calculated using the finished consultant episodes of viral infections characterized by skin and mucous membrane lesions-related admission divided by the mid-year population. We used the chi-squared test to assess the difference between the hospital admission rates between 1999 and 2019. The trend in hospital admissions was assessed using a Poisson model. The correlation between hospital admissions and rates of admission stratified by age and gender was assessed for the duration between 2004 and 2019 using the Pearson correlation coefficient. All analyses were conducted using SPSS version 25 (IBM Corp, Armonk, NY, USA).

In Wales, there was no hospital admission for the diagnostic codes B03 (smallpox) and B04 (monkeypox) during study time. There were two hospital admissions due to B03 in 1999/2000 and 2009/2010, and six hospital admissions due to B04 in 2018/2019 in England. Therefore, B03 and B04 data were excluded from the study calculations.

## 3. Results

The total annual number of hospital admissions for different causes increased by 73.1% from 13,383 in 1999 to 23,169 in 2019, representing an increase in hospital admission rate of 51.9% (from 25.67 (95% CI 25.23–26.10) in 1999 to 38.98 (95% CI 38.48–39.48) in 2019 per 100,000 persons, trend test, *p* < 0.01). 

The most prevalent hospital admissions causes were zoster (herpes zoster), varicella (chickenpox), herpesviral (herpes simplex) infections, and viral warts, which accounted for 26.9%, 23.4%, 18.7%, and 17.6%, respectively (Table 1).

**Figure 1:**
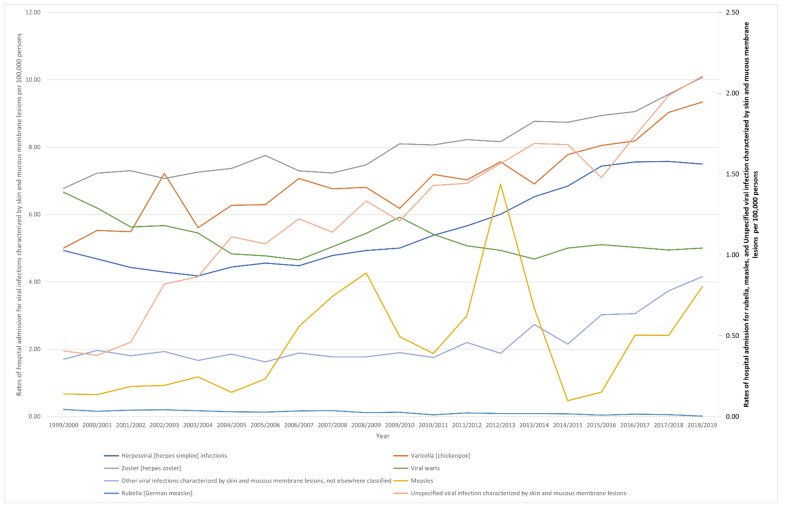
Rates of hospital admission in England and Wales stratified by type between 1999 and 2019. Concerning age group variation for viral infections characterized by skin and mucous membrane lesions hospital admission, during the duration between 1999 and 2019, the age group below 15 years accounted for 43.2% of the total number of viral infections, followed by the age group 15–59 years with 27.2%, the age group 75 years and above with 17.3%, and then, the age group 60–74 years with 12.3%. Rates of hospital admission among patients aged below 15 years increased by 104.8% (from 49.21 (95%CI 47.83–50.59) in 1999 to 100.78 (95%CI 98.88–102.68) in 2019 per 100,000 persons). Rates of hospital admission among patients aged 15–59 years increased by 3.6% (from 15.04 (95%CI 14.61–15.47) in 1999 to 15.58 (95%CI 15.16–15.99) in 2019 per 100,000 persons). Rates of hospital admission among patients aged 60– 74 years increased by 29.0% (from 22.97 (95%CI 21.84–24.10) in 1999 to 29.63 (95%CI 28.52–30.73) in 2019 per 100,000 persons). Rates of hospital admission among patients aged 75 years and above increased by 37.8% (from 54.77 (95%CI 52.45–57.08) in 1999 to 75.49 (95%CI 73.10–77.88) in 2019 per 100,000 persons) (Figure 2).

Through the past two decades, the highest increase in hospital admissions rate was seen in measles, unspecified viral infection characterized by skin and mucous membrane lesions, and other viral infections characterized by skin and mucous membrane lesions, not elsewhere classified, with increases of 4.74-fold, 4.18-fold, and 1.44-fold. Additionally, hospital admissions due to viral infections characterized by skin and mucous membrane lesions rate for varicella (chickenpox), herpesviral (herpes simplex) infections, and zoster (herpes zoster) increased by 86.6%, 52.0%, and 48.7%, respectively. However, the hospital admissions rates for rubella (German measles) and viral warts decreased by 92.4% and 24.9%, respectively (Table 2, Figure 1).

**Figure 3:**
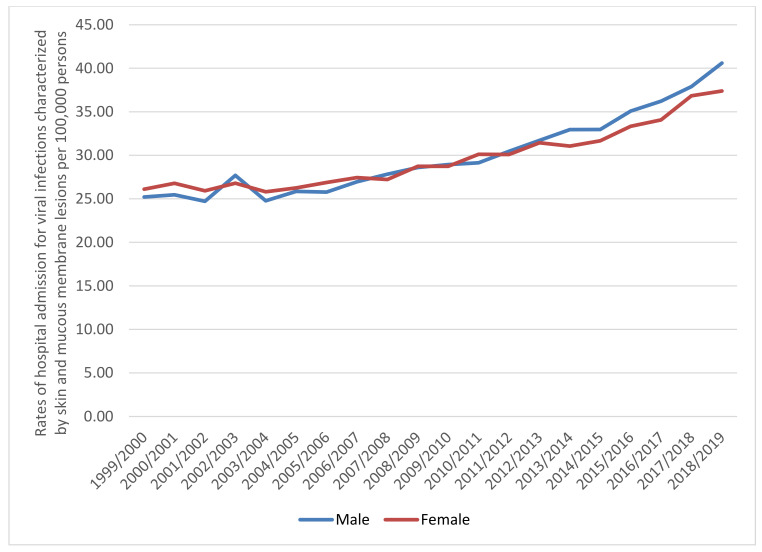
Rates of hospital admission in England and Wales stratified by gender.

A total of 332,262 hospital admission episodes were recorded in England and Wales during the study period. Females contributed to 50.5% of the total number of hospital admission, accounting for 167,827 hospital admission episodes by a mean of 8391 per year. Rates in males increased by 61.1% (from 25.21 (95% CI 24.59–25.82) in 1999 to 40.60 (95% CI 39.87–41.32) in 2019 per 100,000 persons). Rates in females increased by 43.2% (from 26.11 (95% CI 25.49–26.72) in 1999 to 37.40 (95% CI 36.70–38.09) in 2019 per 100,000 persons) (Figure 3).

### 3.1. Admissions Rates and Trends by Gender

**Figure 4:**
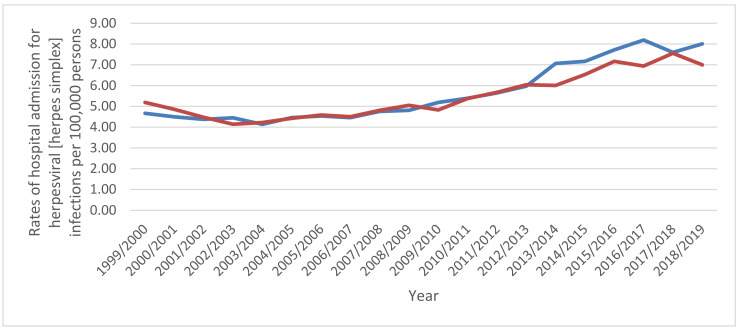

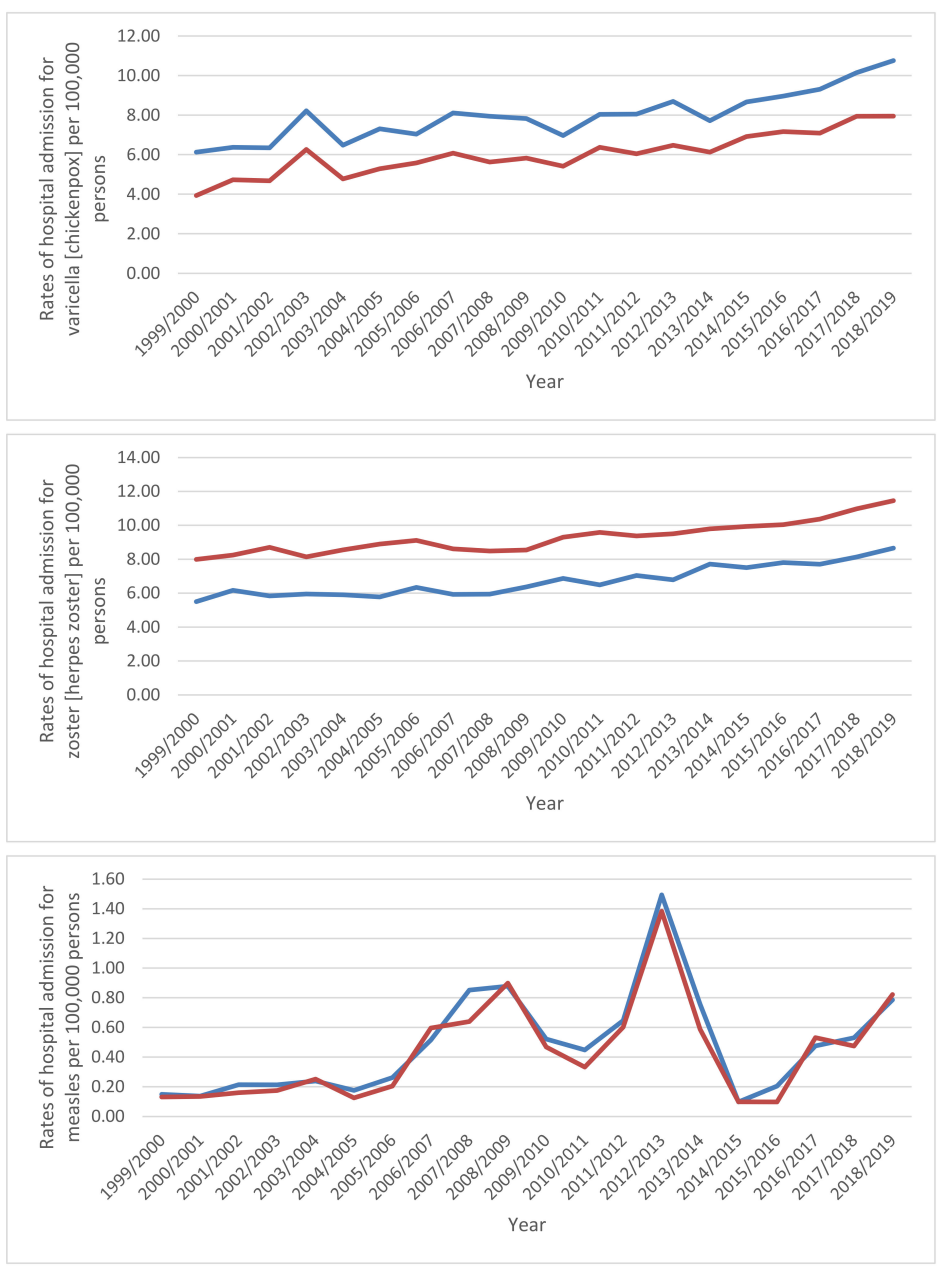

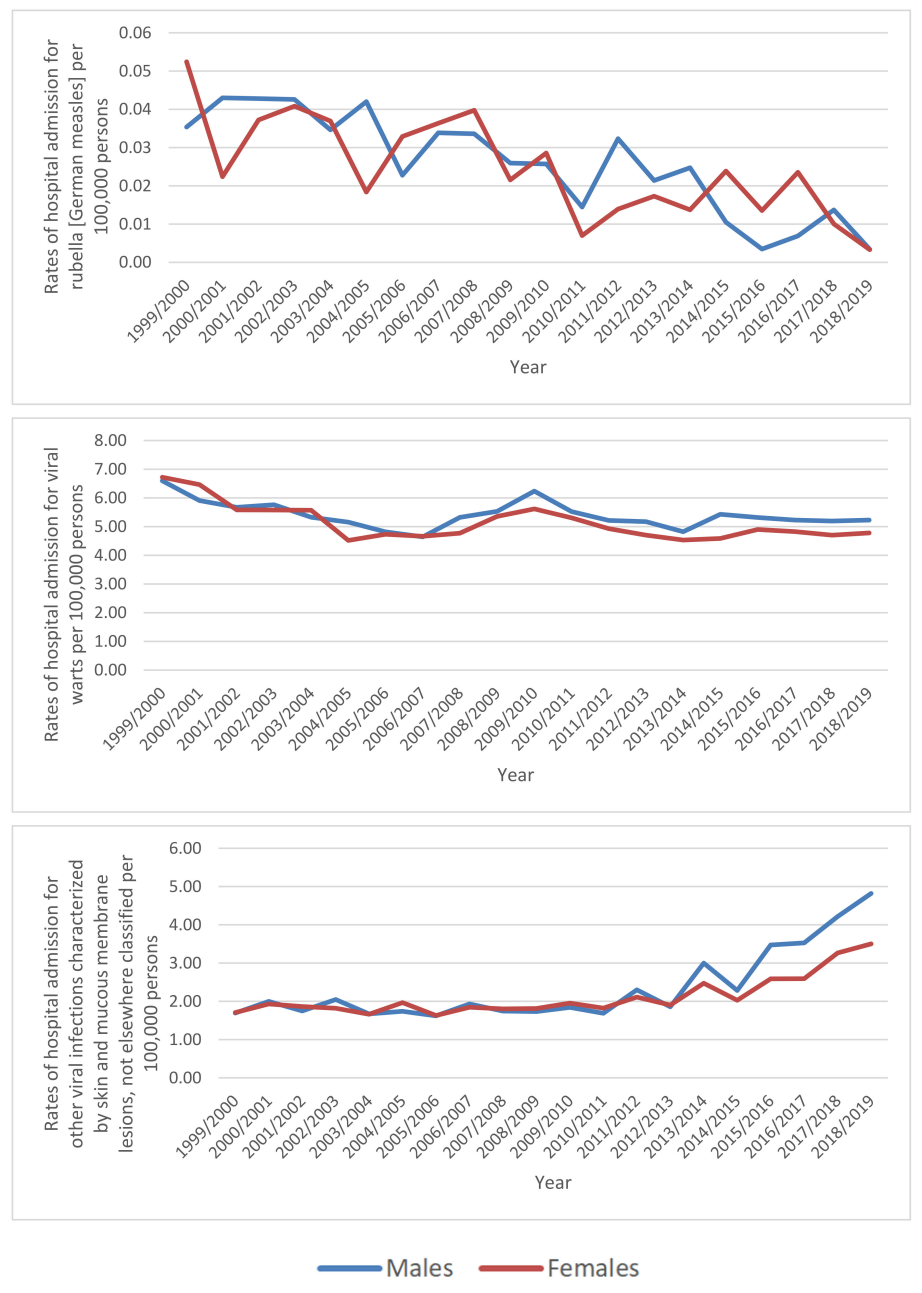
Hospital admission rates in England and Wales stratified by gender.

Hospital admission rates were higher among males compared to females except for zoster (herpes zoster), which were higher among females compared to males (*p* < 0.05) (Figure 4).

### 3.2. Admissions Rates and Trends by Age Group

Several viral infections characterized by skin and mucous membrane lesions-related hospital admissions were seen to be inversely related to age (more prevalent among the age group below 15 years). That includes the following: measles, rubella (German measles), other viral infections characterized by skin and mucous membrane lesions, not elsewhere classified, and unspecified viral infection characterized by skin and mucous membrane lesions. Besides, hospital admissions due to varicella (chickenpox) were more prevalent among the age group: below 15 years, 15–59 years, 75 years and above, and 60–74 years, respectively. Hospital admissions due to herpes viral (herpes simplex) infections were more prevalent among the age group: below 15 years, 75 years and above, 60–74 years, and 15–59 years, respectively. Hospital admissions due to viral warts were more prevalent among the age group: 60–74 years, 75 years and above, 15–59 years, and below 15 years, respectively. However, hospital admissions due to zoster (herpes zoster) were more prevalent among the age group: 75 years and above, 60–74 years, below 15 years, and 15–59 years, respectively.

### 3.3. Correlation between Viral Infection Characterised by Skin and Mucous Membrane Lesions Admissions and Age and Gender

There was strong positive correlation between the rate of hospital admission among patients from all age groups (except the age group 15–59 years) and the hospital admissions for zoster (herpes zoster), varicella (chickenpox), herpesviral (herpes simplex) infections, other viral infections characterized by skin and mucous membrane lesions, not elsewhere classified, and unspecified viral infection characterized by skin and mucous membrane lesions (*p* < 0.01). There was negative correlation between the rate of hospital admission among patients from all age groups (except the age group 64–74 years) and the hospital admissions for viral warts and rubella (German measles) (*p* < 0.05) (Table 3).

There was a strong positive correlation between the rate of hospital admissions among male patients and the hospital admissions for zoster (herpes zoster), varicella (chickenpox), herpesviral (herpes simplex) infections, other viral infections characterized by skin and mucous membrane lesions, not elsewhere classified, and unspecified viral infection characterized by skin and mucous membrane lesions (*p* < 0.01). Similar findings were found among females. There was a negative correlation between the rate of hospital admissions among patients and the hospital admissions for rubella (German measles) (*p* < 0.01) (Table 3).

## 4. Discussion

To the best of our knowledge, this is the first study that has investigated hospital admission trends due to viral infections characterised by skin and mucous membrane lesions in England and Wales during a twenty-year period. The study observed a significant increase in the total annual number of hospital admissions due to viral infections characterised by skin and mucous membrane lesions contributing to an increase in the overall hospital admission rate of 51.9%, with an average of 2.6% per year. The remarkable increase in the rate of hospital admissions among younger population could be connected to the population increase associated with the improvement in life expectancy in both sexes and high annual migration rates between 2005 and 2020 (on average, nearly 0.67% per year) [23]. In England, the total number of hospital admissions reached 16.2 million between 2015 and 2016, a 30% rise from 12.7 million a decade before. Moreover, between 2005 and 2016, the hospital admission rate increased by 30%, a much higher rate than the population, which grew by only 8% [24].

**Figure 5:**
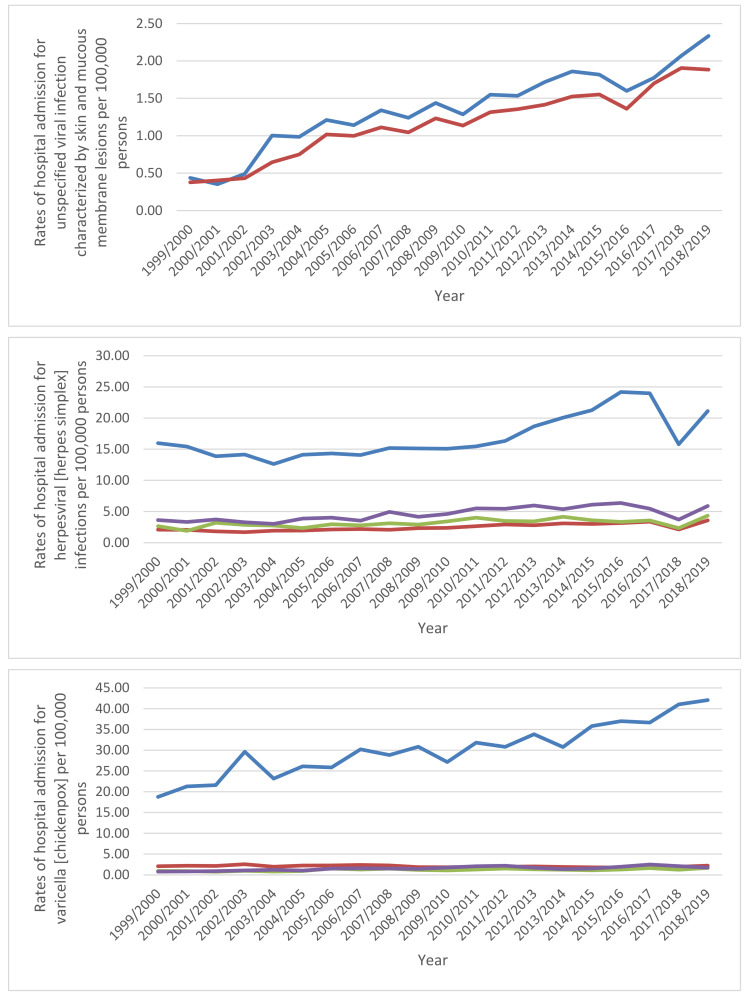

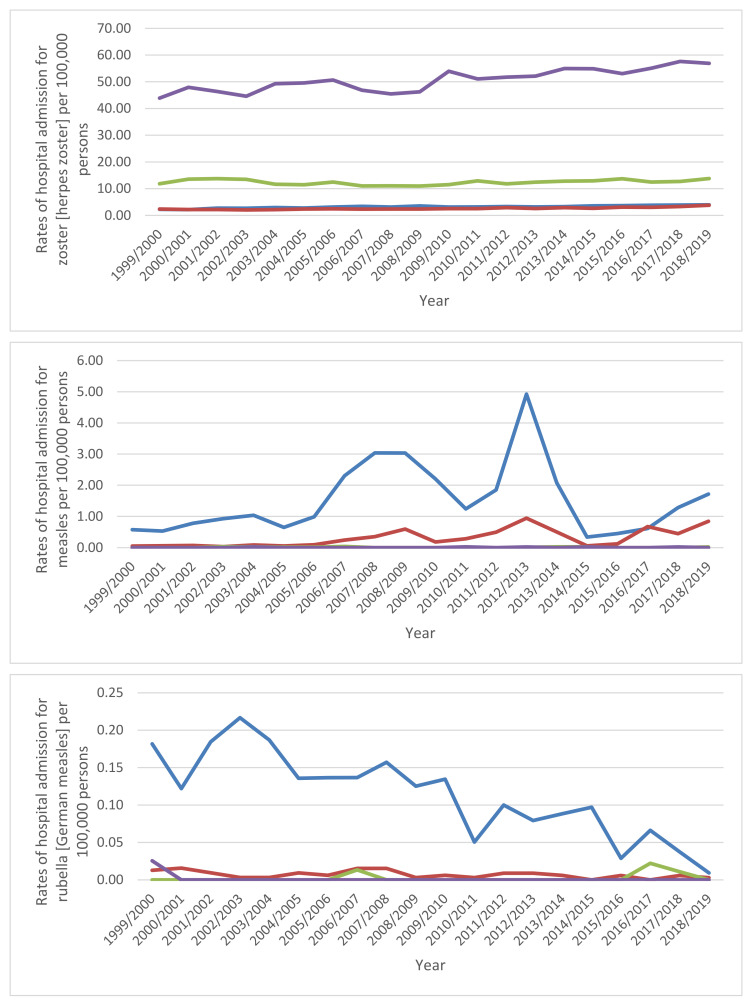

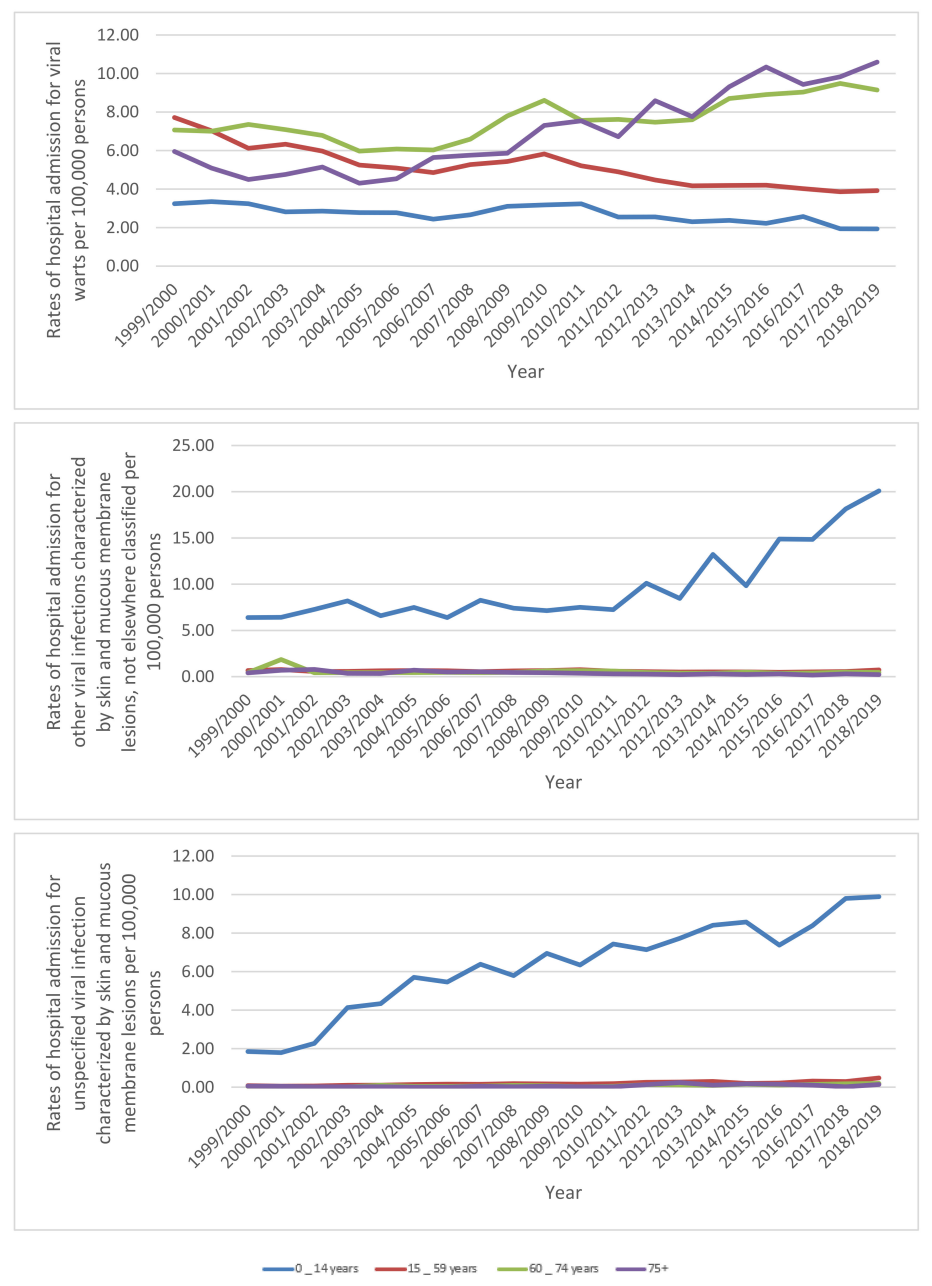
Hospital admission rates in England and Wales stratified by age group.

The current study revealed that herpes zoster (HZ), also known as shingles, and varicella were the most prevalent viral infections characterised by skin and mucous membrane lesions, accounting for 26.9% and 23.4% of total hospital admissions, with an increased rate of hospital admissions by 48.7% and 86.6%, respectively. Shingles is a condition that results from latent varicella-zoster virus (VZV) reactivation, which is responsible for primary infection during childhood (chickenpox). In the UK, the estimated annual number of HZ episodes is about 200,000, and almost one in four people will acquire HZ during their lifetime [25]. In England and Wales alone, the estimated incidence of herpes zoster and varicella is 880 to 960 and 1290 cases per 100,000 people per year, respectively [26,27]. People over 50 years of age are more prone to develop HZ, and the risk is 50% more in those aged 80 years and over [28,29]. The current life expectancy for females and males in the UK is 83.3 and 80.2 years, respectively [29]. The elderly are more prone to various systemic and immunological diseases, making them more vulnerable to HZ and its related complications and in more frequent need of hospital admission [30]. Besides, the process of immune dysfunction with aging, known as immunosenescence, plays a significant role in making the elderly more susceptible to the infection [31]. Regarding varicella, young people are more susceptible to the infection, and the hospital admission rates are higher among them than in the elderly [16,23,32]. These findings are in the same line as our results (Figure 5). This higher rate of hospital admissions among the younger population can be linked to the increased birth rate and high annual migration rates between 2005 and 2020 [23].

Improved life expectancy and the aging population play a critical role in the increasing incidence of HZ in the UK, which explains the highest percentage (26.9%) and the increased rate (by 48.7%) of total hospital admissions due to HZ. These two facts can also explain the increased rates of hospital admission for viral infections characterised by skin and mucous membrane lesions among the study population aged 60–74 years (by 29.0%) and those aged 75 years and over (by 37.8%).

Brisson and Edmunds found that the average incidence rates for zoster and varicella between 1991 and 2000 were 373 and 1291 per 100,000 inhabitants, and the overall hospital admission rates were 4.4 and 4.5 per 100,000 inhabitants, respectively (see Table 2). About 8% and 5% of patients with zoster and varicella, respectively, had underlying immunological diseases [26]. These results are alarming and require prompt action by the government to protect more elderly people (aged 70 years and over, according to the recommendation of the UK’s Joint Committee on Vaccination and Immunisation (JCVI)) with the shingles vaccine and to study the efficacy of the available vaccine more fully [33]. Despite all adults in the UK above 70 being offered vaccines via their general practitioner [34], a previous survey reported that for people aged between 70 and 80 years, vaccine uptake has been low [35]. A study that was conducted in the UK in 2019 reported a low level of self-assessed knowledge about shingles [36]. Additionally, this study showed that GP/nurse vaccine recommendations and vaccine-related self-efficacy construct were important determinants that increase the acceptability of being vaccinated for shingles. On the other hand, non-vaccination was associated with perceived control of the disease, perceived barriers, and previous history of shingles [36].

Our study found a significant decrease (92.4%) in the hospital admission rate related to rubella (German measles), also known as three-day measles [37]. This can be attributed to the MMR vaccine introduced in the UK in 1988, the high effectiveness of the vaccine [38], and the mild nature and limited duration of the disease, since approximately 50% of infected people do not recognise the infection [39]. According to Public Health England data, from 2013 to 2020, there were hardly any confirmed rubella cases in the UK. The extremely low incidence of rubella means that a limited number of women were affected during pregnancy, leading to very few cases of newborns with congenital rubella syndrome (CRS) in the UK [38]. It is worth mentioning that in the duration between January and March 2019, a total of 231 cases of measles were confirmed, which is just three years after the WHO confirmed that the UK was measles free [40].

The authors revealed that patients under 15 years of age accounted for the highest percentage (43.2%) of the total number of hospital admissions due to viral infections characterised by skin and mucous membrane lesions, with a significant increase of 104.8% in the rate of hospital admission. These findings can be attributed to the decline in the infant mortality rate in the UK, which fell from 7 to 3.7 per 1000 live births between 2000 and 2019 [41]. At the same time, this could be due to the change in the behaviour and attitudes towards vaccination among the general population and the improvement in the vaccination pattern. 

Viral infections are common among children, who are susceptible to catching them during pregnancy or delivery, or after birth, since the immune system of premature and newborn babies is immature [41,42]. In addition, it could be that some children and adolescents were admitted to hospital multiple times for treatment of the same or other viral conditions characterised by skin and mucous membrane lesions. 

In the UK in 2020, the estimated population was around 33 million males and 34 million females. This population gender discrepancy has been noticeable over the last 70 years in the UK, where the number of females has always exceeded the number of males. In addition, between 1981 and 2019, life expectancy was lower among men [23]. These statistics explain the slightly higher percentage (50.5%) of hospital admissions due to viral infections characterised by skin and mucous membrane lesions among females during the study period. Additionally, in this study, the hospital admission rate increased at a higher rate among males (by 61.1%) than females (by 43.2%). These findings can explain the slightly higher percentage of hospital admissions between females, despite the large female population. Moreover, the increased hospital admission rate reduced the impact of the high female population on the total number of hospital admissions in the UK. These observations indicate that, over the years, males will become more susceptible to these viral conditions.

The current study found that the hospital admission rates due to viral infections characterized by skin and mucous membrane lesions among males were slightly higher or very close in contrast to females, except for HZ and Varicella, which were significantly higher in females. Our results are in line with those of Lin et al., who found a significant difference by gender in the number of cases and rates of hospital admission due to HZ. They revealed that 59.5% of hospitalised patients were female, with 5.1 more admissions per 100,000 people than males [32]. However, in contrast to our findings, they found no significant difference between males and females regarding Varicella. Another study conducted by Fleming et al. over eight years revealed that of the total number of 14,532 cases of shingles, 59.3% were females and 40.7% males, and the incidence of HZ and varicella was higher in females most of the time than in males [43]. These results can be related to the likelihood of females acquiring and experience recurrence of HZ more than males [44] in addition to the larger population and higher life expectancy in females than males [23]. Females experience significantly higher pain than males due to HZ [44]. Several suggestions have been emerged to explain the gender discrepancy in the incidence of HZ and varicella. Studahl et al. propose that the difference in HZ incidence between females and males may be related to the hormonal differences between genders [45]. Concerning varicella, pregnancy has been identified as a contributory factor for varicella. Besides, women have more frequent contact with children infected with varicella (chickenpox). Thomas and Hall suggest that females tend to seek medical counselling more often than males [46]. Additionally, a previous study by Diaz et al. explored the role for the age and gender in term of vulnerability to infectious diseases and reported that gender plays a role in young patients, where females presented higher infectability and germ aversion than males [47].

Our findings indicated that efforts should be directed towards vaccinating the elderly, particularly females, as the herpes zoster vaccine can reduce the occurrence of shingles for the first time by 50% and reduce the risk of a second episode of the disease [44]. Moreover, efforts should be focused on vaccinating the young population against varicella, particularly females who are more susceptible to acquiring the infection. 

To the best of our knowledge this is the first study to explore the epidemiology of hospital admissions due to viral infections characterised by skin and mucous membrane lesions, without being restricted to specific disease or age group. Our study findings provided comprehensive description for the epidemiology of hospital admissions stratified by age and gender in the past two decades in England and Wales. There are some limitations to this study. First, we could not specifically identify which viral infections characterised by skin and mucous membrane lesions had the greatest influence on the rate of hospital admission among the study population, since some of the viruses can affect several organs independently or simultaneously. Second, this is an ecological study using publicly available data provided by HES and PEDW on the population level, not on the individual level, which restricted our ability to identify the severity of infections, criteria of admission set by hospitals, reimbursement policies, and the risk factors associated with viral infections of the skin and mucous membranes, including comorbidities and immunodeficiency.

We did not have detailed information on the sub-categories reported under the ICD code B09 “Unspecified viral infection characterized by skin and mucous membrane lesions”, which might lead to the possibility of underreporting of hospital admission data. Health technologies advancement in the past 20 years is an important factor that has improved diseases diagnostic process and quality, which could have contributed to the increase in the admission rates for specific diseases (increased reporting capabilities of the healthcare system). On the other hand, during the past decade, vaccination programs have contributed markedly to the decrease in the prevalence of a wide range of diseases and decreased the severity of them and the need for hospitalisation for them. Furthermore, we used only ICD-10 codes in Chapter I (B00–B09) only. Many skin and mucous membrane legions due to viral infection might be coded in other chapters by organ system such as the ICD-10 codes K12.3 for oral ulcer, N76.5 for vaginal ulcer, or N76.6 for vulva ulcer, which are not included in our findings.

## 5. Conclusions

The most common hospital admission causes were zoster (herpes zoster), varicella (chickenpox), herpesviral (herpes simplex) infections, and viral warts. Our study demonstrates an evident variation in hospital admissions of viral infections characterized by skin and mucous membrane lesions based on age and gender. Efforts should be directed towards vaccinating high-risk groups, particularly the elderly and females. Further observational epidemiological studies are needed to identify other factors associated with increased hospital admission rates.

## Figures and Tables

**Figure 2:**
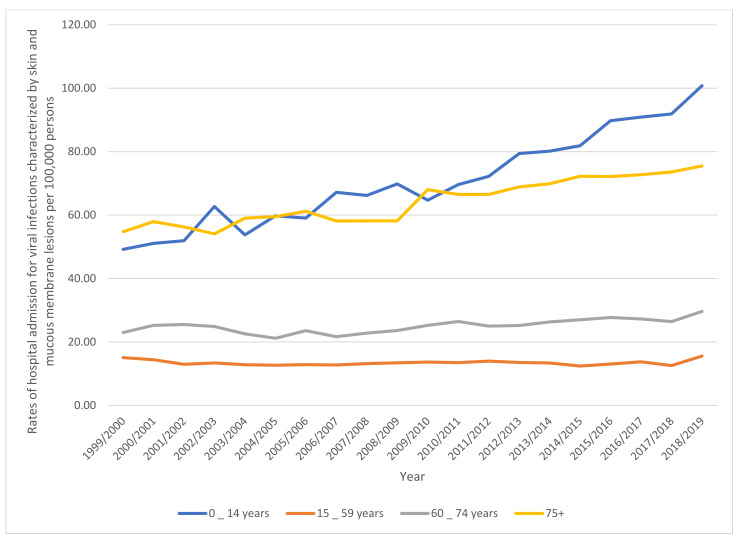
Rates of hospital in England and Wales stratified by age group.

**Table 1:** Percentage of viral infections characterized by skin and mucous membrane lesions hospital admission from total number of admissions per ICD code during the study period.

ICD Code	Description	Percentage from Total Number of Admissions
B02	Zoster (herpes zoster)	26.9%
B01	Varicella (chickenpox)	23.4%
B00	Herpesviral (herpes simplex) infections	18.7%
B07	Viral warts	17.6%
B08	Other viral infections characterized by skin and mucous membrane lesions, not elsewhere classified (orthopoxvirus infections, Molluscum contagiosum, Exanthema subitum, Erythema infectiosum, Enteroviral vesicular stomatitis with exanthema, and Enteroviral vesicular pharyngitis)	7.5%
B09	Unspecified viral infection characterized by skin and mucous membrane lesions	4.3%
B05	Measles	1.5%
B06	Rubella (German measles)	0.1%

ICD International Statistical Classification of Diseases system.

**Table 2:** Percentage change in the hospital admission rates from 1999 to 2019 in England and Wales.

Diseases	Rate of Diseases in 1999 per 100,000 Persons (95% CI)	Rate of Diseases in 2019 per 100,000 Persons (95% CI)	Percentage Change from 1999 to 2019
Herpesviral (herpes simplex) infections	4.93 (4.74–5.12)	7.50 (7.28–7.72)	52.0%
Varicella (chickenpox)	5.00 (4.81–5.20)	9.34 (9.09–9.58)	86.6%
Zoster (herpes zoster)	6.78 (6.55–7.00)	10.08 (9.82–10.33)	48.7%
Measles	0.14 (0.11–0.17)	0.80 (0.73–0.88)	474.4%
Rubella (German measles)	0.04 (0.03–0.06)	0.00 (0.00–0.01)	−92.4%
Viral warts	6.66 (6.44–6.88)	5.00 (4.82–5.18)	−24.9%
Other viral infections characterized by skin and mucous membrane lesions, not elsewhere classified	1.70 (1.59–1.81)	4.15 (3.99–4.32)	144.1%
Unspecified viral infection characterized by skin and mucous membrane lesions	0.41 (0.35–0.46)	2.11 (1.99–2.22)	418.0%

**Table 3:** Correlation between viral infection characterised by skin and mucous membrane lesions admissions and age and gender between 2004 and 2019.

Type of Viral Infection Characterised by Skin and Mucous Membrane Lesions	Zoster (Herpes Zoster)	Varicella (Chickenpox)	Herpesviral (Herpes Simplex) Infections	Viral Warts	Other Viral Infections Characterized by Skin and Mucous Membrane Lesions, Not Elsewhere Classified	Unspecified Viral Infection Characterized by Skin and Mucous Membrane Lesions	Measles	Rubella (German Measles)
Age								
Below 15 years	0.930 **	0.953 **	0.925 **	−0.570 **	0.851 **	0.937 **	0.418	−0.831 **
15–59 years	0.135	0.015	0.154	0.483 *	0.280	−0.046	0.202	−0.225
64–74 years	0.824 **	0.667 **	0.809 **	−0.088	0.742 **	0.575 **	0.107	−0.770 **
75 years and above	0.957 **	0.787 **	0.916 **	−0.466 *	0.763 **	0.878 **	0.295	−0.850 **
Gender								
Males	0.958 **	0.924 **	0.953 **	−0.431	0.919 **	0.888 **	0.357	−0.840 **
Females	0.963 **	0.915 **	0.952 **	−0.410	0.908 **	0.882 **	0.359	−0.879 **

* *p* < 0.05; ** *p* < 0.01.

## Data Availability

Publicly available datasets were analyzed in this study. This data can be found here: http://www.infoandstats.wales.nhs.uk/page.cfm?pid=41010&orgid=869 (accessed on 20 October 2021).
